# A Lying-In Hospital for Leeds

**Published:** 1897-10-16

**Authors:** 


### A Lying-in Hospital for Leeds.

An appeal is being made in Leeds for funds with which to rebuild the Hospital for Women and Children and add to it an in-patient maternity department. We need hardly say with what careful thought any such proposal should be entered upon. There can be no doubt that the hospital system of England remains sadly incomplete, in view of the practical absence of in-patient accommodation for maternity cases. Until within quite recent years sad experience showed the great dangers which attached to all lying-in institutions as then conducted, and the propriety of allowing even such lying-in hospitals as existed to continue their operations was seriously disputed, so frequent and so deadly were the outbreaks of puerperal fever within their walls. By the use of antiseptics, that has now become a story of the past, and possibly the safest place in which a woman can at the present day face the perils of childbirth is in a well-conducted hospital devoted to the reception of lying-in cases. The attachment of an in-patient maternity department to a hospital in which a considerable variety of cases must of necessity be received is, however, quite another matter, especially when that hospital is one for children, among whom infectious cases are peculiarly apt to occur. It must be remembered that in regard to lying-in hospitals, even such as stand isolated and by themselves, we are only just out of the wood, and that the attachment of such institutions to hospitals which are in any way "general," in the sense of having to receive all sorts of cases, is a grave experiment. We hope that if the experiment is to be tried, every precaution will be taken, and that, as in the Rotunda at Dublin, the lying-in and the gynæcological departments will be in separate buildings.

The association of women with children in so many institutions which are looked upon as "special hospitals," is a matter which requires looking into and arranging afresh, and should on this occasion be carefully reconsidered. So far as "special" treatment in "special" institutions is concerned, "women" and "children" have absolutely nothing to do with each other, and their association really seems to be a relic of the times when ladies were so modest that they could only whisper to grey hairs, and the treatment of children's diseases was such a mystery that it could only be learned by "experience." But we have changed all that, and it seems high time that children with their febrile ailments should be separated from midwifery and gynæcology, both of which, so far as in-patient treatment is concerned, are matters of active surgery.

